# Mouse Genome Informatics (MGI): latest news from MGD and GXD

**DOI:** 10.1007/s00335-021-09921-0

**Published:** 2021-10-26

**Authors:** Martin Ringwald, Joel E. Richardson, Richard M. Baldarelli, Judith A. Blake, James A. Kadin, Cynthia Smith, Carol J. Bult

**Affiliations:** The Jackson Laboratory, Bar Harbor, ME USA

## Abstract

The Mouse Genome Informatics (MGI) database system combines multiple expertly curated community data resources into a shared knowledge management ecosystem united by common metadata annotation standards. MGI’s mission is to facilitate the use of the mouse as an experimental model for understanding the genetic and genomic basis of human health and disease. MGI is the authoritative source for mouse gene, allele, and strain nomenclature and is the primary source of mouse phenotype annotations, functional annotations, developmental gene expression information, and annotations of mouse models with human diseases. MGI maintains mouse anatomy and phenotype ontologies and contributes to the development of the Gene Ontology and Disease Ontology and uses these ontologies as standard terminologies for annotation. The Mouse Genome Database (MGD) and the Gene Expression Database (GXD) are MGI’s two major knowledgebases. Here, we highlight some of the recent changes and enhancements to MGD and GXD that have been implemented in response to changing needs of the biomedical research community and to improve the efficiency of expert curation. MGI can be accessed freely at http://www.informatics.jax.org.

## Introduction to Mouse Genome Informatics

As the cost of genome sequencing continues to decrease and as genome editing technologies become widely adopted, the laboratory mouse is more important than ever as a model system for understanding the biological significance of human genetic variation and for research needed to validate and safely advance the emerging practice of genomic medicine.

Mouse Genome Informatics (MGI) is a community genome knowledgebase resource focused on supporting investigations into human biology and disease through the integration of genetic, genomic, and biological data for the laboratory mouse. MGI comprises multiple databases that share common infrastructure and/or common data standards: Mouse Genome Database (MGD) (Blake et al. 2021), Gene Expression Database (GXD) (Baldarelli et al. 2021), Mouse Models of Human Cancer database (MMHCdb) (Krupke et al. 2017), CrePortal (Perry et al. 2021), and the International Mouse Strain Resource (IMSR) (Eppig et al. 2015b). In this report, we focus on the latest advancements for the two major database components of MGI: MGD and GXD.

The uniqueness of the resources available from MGI is rooted in our emphasis on deep *data integration* which is enabled by the rigorous semantic standards applied to the biological annotations represented in MGI’s contributing databases. MGI search capabilities extend far beyond browsing or keyword searches because they allow researchers to combine multiple parameters and concepts in a single search. For example, a query such as “What genes on chromosome 17, studied in mouse models of Spina bifida, are expressed in the neural tube and involved in signaling?” can be answered with a single search at MGI even though the data/information about genome location and feature type, disease/developmental disorder association, and function needed to answer this question with precision comes from many different sources. In contrast, search engines such as PubMed and Google lack the rigor of ontological standardization as well as the underlying information system architecture to support this type of data and knowledge integration. To answer a question like the one above outside of the MGI environment, multiple searches would be required, followed by the daunting and time-consuming tasks of sorting through and collating and quality checking a large resulting data set. The value of MGI as a core community knowledgebase is rooted in our constant extension and integration of new data types, and our support for comparative views of knowledge about human and mouse biology. We continually adapt our operational processes to accommodate new types of experimental data and refine our knowledgebase to reflect evolving understanding of biological systems. Innovation is reflected in our scalable solutions to data acquisition, deep data integration, and execution of FAIR (Findable, Accessible, Interoperable, Reproducible) principles of data management (Wilkinson et al. 2016).

Programmatic access to MGI is supported by MouseMine (Motenko et al. 2015), a data warehouse built with InterMine (Smith et al. 2012). MouseMine provides computational users with powerful features such as customizable queries and reports, lists which can be saved from query results or used to drive subsequent queries, built in enrichment analysis, and interconnections with mines for other model organisms. MouseMine is updated weekly from MGI and includes core data such as the unified mouse genome feature catalog, biological annotations including gene function, phenotype and disease associations, and gene expression data. As well, MouseMine includes the complete genomes (assemblies and gene models) of 19 mouse strains. A comprehensive API provides programmatic access to all features, data, and tools in MouseMine, significantly expanding FAIR access to MGI data.

One of the hallmarks of MGI is the expert curation which underlies robust data integration of knowledge from the peer-reviewed scientific literature and other sources. The mouse literature is expanding at a rate of over 80,000 publications in PubMed per year, but only a fraction of these publications is relevant to the MGI mission. For example, over the years 2017–2020, MGI curators reviewed an average of 31,197 papers per year and selected (aka, triaged) 14,820 of those papers for detailed curation. MGI-relevant publications are published in approximately 900 scientific journals—with most papers coming from approximately 120 journals. Identifying relevant articles requires searching full text, as many of these publications do not mention mouse in the title, abstract, or keywords. A recent enhancement to MGI’s literature triage process is the implementation of a machine learning classifier applied to the full text of each article to decide if it is appropriate for at least one of the MGI knowledgebase resources. The performance metrics of precision, recall, and negative predictive value (NPV) for the classifier are 0.85, 0.90, and 0.90, respectively. An NPV of 0.90 means that 90% of the predictions that an article is not relevant to any of the databases that make up MGI are correct and only 10% are false negatives (i.e., incorrectly discarded). The application of machine learning to this critical step in the curation process allows curators to efficiently ‘discard’ papers that are not relevant, thus saving the hundreds of hours of manuscript review time.

On average, MGI receives 7.5 million page views per year and is accessed by a user base of over 400 thousand individuals that use MGI 1 to 9 times per year. More than 14 thousand users access the site at least 10 times per year. These usage data underestimate the total MGI user base as many researchers access MGI annotations and information from other widely used data resources including, but not limited to, the Alliance of Genome Resources (Alliance of Genome Resources 2020), the Rat Genome Database (RGD) (Smith et al. 2020b), the National Center for Biotechnology Information’s (NCBI) Gene resource (Brown et al. 2015), the International Mouse Phenotyping Consortium (IMPC) (Munoz-Fuentes et al. 2018), the Ensembl genome browser (Newman et al. 2018), UCSC Genome Browser’s VisiGene (Kuhn et al. 2007), SciCrunch (Bandrowski et al. 2015), the Protein InteraCtion KnowLedgebasE (PICKLE) (Dimitrakopoulos et al. 2020), the Online Gene Essentiality database (OGEE) (Gurumayum et al. 2021), Bio Gene Portal System (BioGPS) (Ringwald et al. 2012), GlyGen (York et al. 2020), JAX Synteny Browser (Kolishovski et al. 2019), Mutant Mouse Resource and Research Center (MMRRC) (Amos-Landgraf et al. 2021), European Mutant Mouse Archive (EMMA) (Hagn et al. 2007), Gene Ontology Consortium (Gene Ontology 2015), and the Online Mendelian Inheritance in Man (OMIM) knowledgebase (Hamosh et al. 2021). Annotations from MGI are also central to the development and application of machine learning and semantic reasoning methods for the prediction of mouse gene function and phenotypes for such projects as GeneWeaver (Baker et al. 2012), Functional Networks of Tissues in Mouse (FNTM) (Goya et al. 2015), Phenodigm (Smedley et al. 2013), and the Monarch Initiative (Shefchek et al. 2020).

## Mouse Genome Database (MGD)

MGD is the community model organism database for the laboratory mouse and is a primary resource for biological reference data related to mouse genes and other genome features, functional annotations, phenotypes, and disease models with a strong emphasis on the relationship of these data to human biology and disease. Each of these areas is described below. The resources and annotations for which MGD serves as the authoritative source are shown in Table 1. The primary target user communities for MGD include basic scientists and translational/clinical researchers using mouse as a model organism, computational biologists, and bioinformatics resource development groups. In addition to being a major component of the MGI information system, MGD is a founding member of the Alliance of Genome Resources (Alliance of Genome Resources 2019; Alliance of Genome Resources Consortium 2019). The Alliance brings together expertly curated annotations from multiple model organisms and human into a single information portal, greatly simplifying the comparison of biological knowledge available for the genomes of diverse model organisms.

**Table 1:** Resources and annotations for which MGD serves as the authoritative source

Data type or resource	Maintained as	Explanation
Unified mouse genome feature catalog	Catalog of genome features with chromosome location, persistent identifiers, cross-links to other provider identifiers and sequences; Table of the distribution of equivalent genome features across different strains of mice	MGD integrates computational gene predictions from GenCode/Ensembl and NCBI into a single, comprehensive, non-redundant resource. The catalog is the source of mouse genome feature annotations for the Alliance of Genome Resources
Gene Ontology (GO) annotations for mouse	Associations between mouse genes and GO terms incorporating contextual data for function assertions from multiple biomedical ontologies	MGD provides primary curation from the scientific literature and other mouse annotation groups and distributes the definitive set mouse GO annotations
Mammalian Phenotype (MP) Ontology	Ontology of defined phenotype terms and relationships	MGD develops vocabulary terms with community input and distributes MP via multiple ontology resource sites and the MGD FTP site
Mammalian Phenotype Ontology (MP) annotations	Associations between mouse genotypes and MP terms	MGD provides primary curation and integration with alleles, strains, and genotypes
Mouse models of human diseases	Associations between mouse genotypes and human disease terms	MGD provides primary curation with ongoing coordination with Disease Ontology, OMIM, and NCBI for human gene-disease associations
Symbols and names for genes & genome features	Official nomenclature for genome features; synonyms; nomenclature history	MGD is the web host for nomenclature rules set by International Committee on Standardized Genetic Nomenclature for Mice; nomenclature is coordinated with nomenclature groups for human and rat
Symbols and names for mutant alleles, transgenes, and genome rearrangements	Comprehensive catalog of mutations; persistent identifiers; descriptions of mutant construction and inheritance	MGD makes primary assignments; coordinates with many mutagenesis projects
Strain nomenclature	Catalog of mouse strains with persistent identifiers	MGD provides nomenclature and unique, permanent identifiers to multiple resources

### Unified gene catalog, variants, and orthology

The foundation for biological annotations (i.e., function, phenotype, expression) in MGI is the MGD unified gene catalog (Zhu et al. 2015). The unified gene catalog is generated by combining the mouse genome annotations generated by GenCode/Ensembl (Frankish et al. 2021) and NCBI (https://www.ncbi.nlm.nih.gov/genome/annotation_euk/process/) into a canonical, non-redundant catalog of mouse genes and other genome features for the laboratory mouse. We create and maintain the unified gene catalog as a consensus view of mouse genome annotation as many predicted genes are unique to a particular genome annotation pipeline.

The unified gene catalog is generated using a semi-automated analysis pipeline that determines equivalency of two gene predictions (annotations) based on genome coordinate overlap. We use the feature join (“fjoin”) algorithm to determine coordinate overlap (Richardson 2006). The inputs for fjoin are files of genome coordinates of predicted features from two different sources that are in General Feature Format (GFF3; http://www.sequenceontology.org/gff3.shtml). The fjoin algorithm is designed to determine overlaps of genome coordinates very efficiently; the comparison of two genome annotation inputs with hundreds of thousands of annotated features takes only minutes to perform. Any genome feature that has genome coordinates can be used as input. fjoin outputs sets of features that have coordinate overlaps and those without overlaps. Genes with coordinate overlap are considered “equivalent.” Equivalent genome features are not required to have identical structures and the degree to which features need to overlap to be considered equivalent is an adjustable parameter. We have recently updated all genome coordinates to the latest mouse genome assembly (GRCm39, GenBank accession: GCA_000001635.9) and integrated all genome annotations from GenCode/Ensembl and NCBI into the current MGD unified gene catalog.

**Fig. 1:**
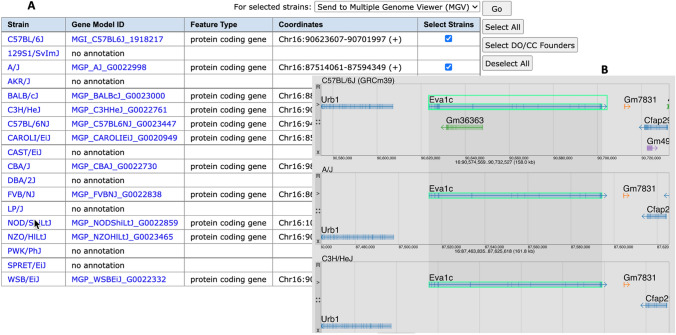
**A** Tabular summary of the *Eva1c* gene (MGI:1918217) annotation across multiple strains of mice with three strains selected. **B** Screenshot of the Multiple Genome Viewer showing the *Eva1c* and surrounding annotations for the strains selected in the strain annotation distribution table

Another recent enhancement to MGD’s representation of genome features is the addition of annotations available from Ensembl for the genomes of 16 inbred mouse strains (Lilue et al. 2018) and two wild-derived strains (CAROLI/EiJ and PAHARI/EiJ) (Thybert et al. 2018). From MGD, the strain distribution of genome annotations is provided in tabular form allowing researchers to quickly identify genes that are in some strains but not others (Fig. 1A). To support visualization and interaction with multiple annotated genomes at one time, we also developed the Multiple Genome Viewer (MGV) (Richardson et al. 2021). MGV is a highly configurable visualization tool that is integrated with all the biological annotations of mouse genes for phenotype, function, and disease from MGD (Fig. 1B).

#### Sequence context for phenotypic alleles

The representation of the sequence context of variants in the mouse genome is another major focus for MGD curation efforts. Data for the sequence context of large-scale, high-throughput variants for the laboratory mouse are readily available from the European Variation Archive (EVA; https://www.ebi.ac.uk/eva/). For the set of variants from EVA available in MGD, we provide unique search and data display interfaces that support the identification of nucleotide variants that occur in some strains but not others. In contrast to high-throughput variants, the sequence context for mutant alleles of mouse genes reported in the scientific literature are largely available as free text. The lack of a standard syntax for representing these variants means they are difficult to search for or to use in computational analyses. MGD curators are systematically reviewing text descriptions available for tens of thousands of mutant mouse alleles associated with phenotypes and are converting them from text to Human Genome Variation Society (HGVS) standard notations (den Dunnen 2017). For example, the mutation details in MGD currently for the ENU-induced *Megf8*^*b2b288Clo*^ allele (MGI:5311364) are as follows: “The causative molecular lesion for the cardiovascular phenotypes is a A to T single point mutation at position 3641 of the cDNA (c.A3641T) (RefSeq NM_001160400). This is predicted to alter an arginine residue to an isoleucine at position 1214 (p.N1241I) in the encoded protein.” This allele is now also represented in standard HGVS notation (NC_000073.6:g.25342303A > T) making it far easier to precisely locate the mutation on the mouse reference genome assembly and to use the variation as input into algorithms such a JANNOVAR (Jager et al. 2014) or the Ensembl Variant Effect Predictor (McLaren et al. 2016) to predict the functional consequences of the variation. Curated sequence notations for mouse phenotypic alleles are displayed on gene detail pages for the mouse at the Alliance of Genome Resources website (https://alliancegenome.org) and are also available for download from the Alliance.

#### Orthology

MGD’s representation of orthology for genome features between mouse and other vertebrate species provides the basis for comparing biological knowledge and information between mouse and other vertebrate species. Orthology is central to many of the user interfaces supported by MGD including the Multiple Genome Viewer (see Richardson et al., this issue) and the Human–Mouse Disease Connection (HMDC) (Eppig et al. 2015a). We recently migrated our representation from NCBI’s Homologene (Coordinators 2016) to the orthology assertions maintained by the Alliance of Genome Resources which are based on the DIOPT algorithm (Hu et al. 2011). As the ortholog data from the Alliance are currently limited to human, mouse, rat, zebrafish, fly, worm, and yeast, we use HGNC’s HCOP as a source of orthology for vertebrate species not yet in the Alliance set, including chimp, dog, and some agricultural species (Eyre et al. 2007; Yates et al. 2021).

### Functional annotation

MGD is the leading source of annotations about the function of mouse protein-coding genes and functional RNAs, providing these data in a variety of formats to computational biologists and bioinformaticians and to general users in human readable formats through the MGI web interface, the Gene Ontology Consortium (GOC) AmiGO portal (Carbon et al. 2009), and the Alliance of Genome Resources (Alliance) (Alliance of Genome Resources 2019; Alliance of Genome Resources Consortium 2019). More than 32,000 mouse genome features (protein-coding and non-coding genes) in MGD have at least one functional annotation with almost 40% of these annotations based on direct experimental evidence. Nearly 500,000 curated GO annotations are available for mouse from MGD.

The annotation standards afforded by the Gene Ontology have been critical to robust comparisons of gene function across many organisms and to gleaning biological insights from the analysis of genome scale data (The Gene Ontology 2019). To make functional annotations using the Gene Ontology even more useful for representing biological systems, MGD curators have been working with the Gene Ontology Consortium to develop Causal Activity Models based on GO (GO-CAM) (Thomas et al. 2019). GO-CAM are models of biological systems and pathways that are generated by linking together multiple GO annotations in a semantically structured manner using the Relation Ontology (Smith et al. 2005). Over 300 GO-CAM models are currently available for mouse currently (https://geneontology.cloud/groups/MGI). Although not widely used by the research community currently, GO-CAMs are an important emerging informatics technology that will contribute to the development of data analysis methodologies that leverage expert curation for computational data integration and hypothesis generation.

### Phenotype and disease annotation

The Mammalian Phenotype Ontology (MP) (Smith and Eppig 2012) is essential to the integration of phenotype data in MGD. MP is a structured vocabulary whose development and continued maintenance is centered at MGD with community input. The MP supports general and granular phenotype knowledge and allows robust searching and retrieval for web-based users as well as computational users. Each of the more than 13,000 vocabulary terms in the MP has an accession ID, name, synonyms, definition, and a reference for the definition. Terms are organized hierarchically from general to specific, so phenotypes can be annotated at the finest level of granularity to reflect current knowledge. Over 360,000 MP annotations have been made in MGD and are associated with 54,177 mutations in 20,009 genes, transgene, and other markers. The MP has been adopted widely including by the Rat Genome Database (Smith et al. 2020b), the International Mouse Phenotyping Consortium (Munoz-Fuentes et al. 2018), and the Monarch Initiative (Mungall et al. 2017).

To enable the comparison of phenotype terms between mouse and human, we collaborate with the developers of the Human Phenotype Ontology (HPO) (Kohler et al. 2019) to generate mappings of phenotype terms between the two ontologies. We are collaborating to ensure that the definitions for the terms in the ontologies are correct and that they are comparable for equivalent terms. As a result, MP and HPO maintain the terminologies in common use among mouse geneticists and human clinicians, respectively, while sharing a common equivalence axiom that allows computational mapping of equivalent terms. This mapping, in turn, enables comparisons between phenotype profiles for mutant mice and for humans with genetic conditions to select the best mouse model or to select the best set of candidate genes.

While many of the nearly 13,000 phenotype terms in the MP have terms in the HPO that parallel each other in name and meaning, other terms share a common meaning but have different names. As well, some concepts are unique to one species or the other, and some can be “matched” or partially matched but are conceptually difficult to establish equivalencies. For example, cataract is a shared term in mouse and human with identical use, whereas absent eyelids (mouse) is equivalent to ablepharon (human), but the specific terms used differ. Complex examples include descriptions of abnormalities of the prostate gland because the anatomical structure is different in human and mouse, even though the organ performs a similar function in the two organisms. A particularly complex area for phenotype comparison is behavior as equivalence of behavioral phenotypes in mouse and human can be difficult to establish. To date, approximately 80% of MP and 50% HPO have been reviewed and agreed on as to Ontology Web Language (OWL) pattern equivalence axioms. MGI, HPO, and the Alliance continue to work collaboratively to develop equivalence axioms across multiple species as members of the POTATO (Phenotype Ontologies Transversing all the Organisms) community (https://zenodo.org/record/2382757).

### Disease models

Providing access to published and potential mouse models of human disease is one of the core functions of MGD. The Human–Mouse: Disease Connection (HMDC) (Eppig et al. 2015a) was developed specifically for this purpose. Mouse mutation, and phenotype and disease model data are integrated with human gene-to-disease relationships from the National Center for Biotechnology Information (NCBI) and Online Mendelian Inheritance in Man (OMIM) and with human disease-to-phenotype relationships from the Human Phenotype Ontology (HPO). One of the significant enhancements to HMDC is the use of the Disease Ontology (DO) for standard disease annotations (Bello et al. 2018; Schriml et al. 2019). Previously, most of the disease annotations in MGD and HMDC were based on OMIM phenotype terms. The DO provides a much broader disease terminology for disease annotation but retains cross-links to other terminologies, including OMIM.

**Fig. 2:**
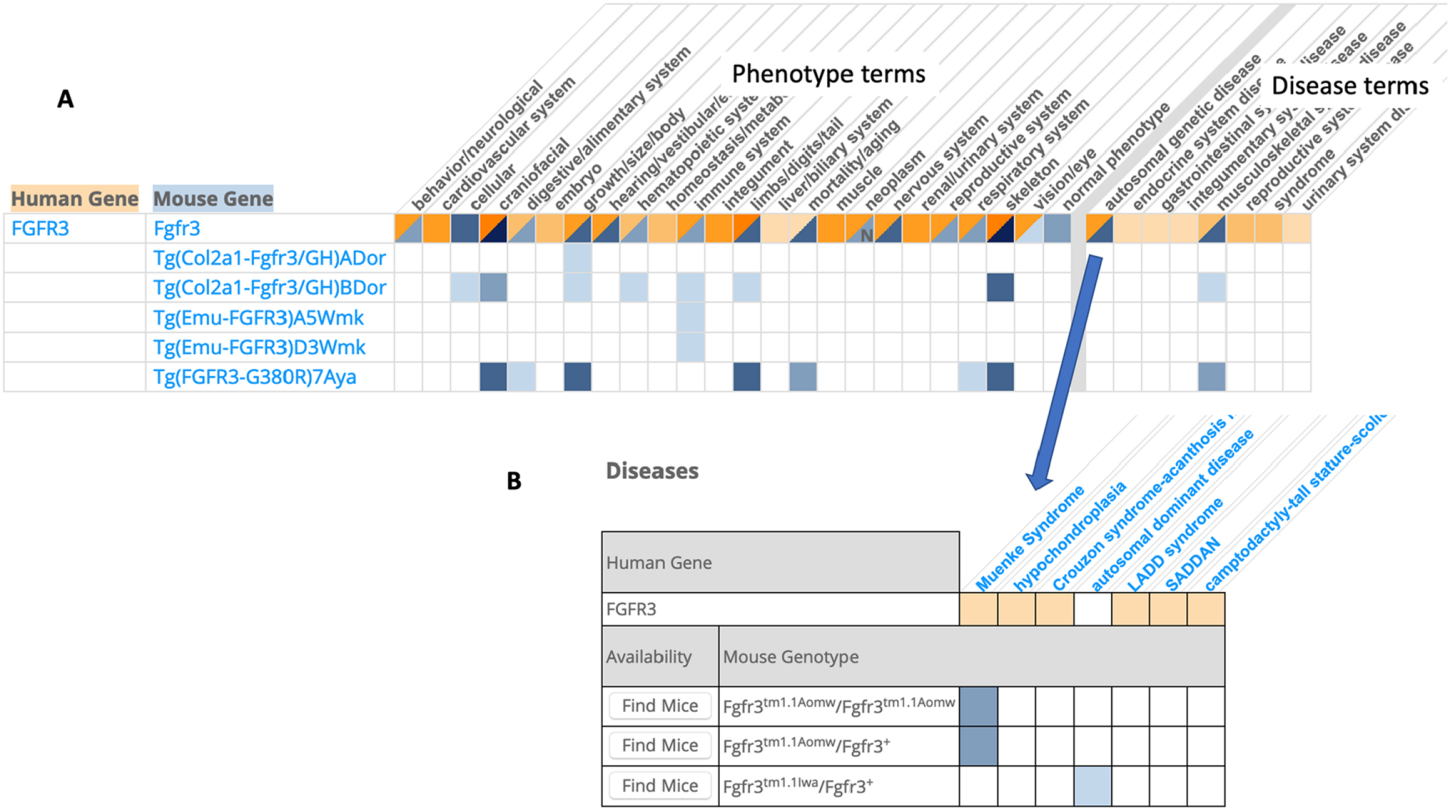
Screenshot from MGD’s Human–Mouse Disease Connection (HMDC) **A** Results of search for the gene *Fgfr3* (MGI:95524) showing the ribbon format overview of phenotype term annotation similarities of mouse and human. Cells with orange indicate annotations for the human gene for a phenotype term. Cells with blue shading indicate annotations for a mouse gene for a phenotype term. Cells with both orange and blue indicate a phenotype term shared by mouse and human. Depth of color indicates the number of published annotations. Mouse genes starting with “Tg” represent transgenic mice carrying either the mouse or human *Fgfr3* gene. **B** Arrow indicates the results of clicking on a cell for a disease term showing specific disease annotations at the gene level for human and at the genotype level for the mouse. The “Find Mice” button is a link to the International Mouse Strain Resource (IMSR) (Eppig et al. 2015b) which lists the availability of mouse models from repositories around the world

One of the major benefits to the HMDC is that researchers can search by human or mouse gene symbols, human or mouse phenotype terms, or human disease terms. The search results are displayed with color coding that indicates how similar the phenotypes and disease annotations are between mouse and human for a given gene (Fig. 2). While gene-to-disease relationship are useful, they can also be misleading as the phenotypes observed for an allelic variant of a gene can differ greatly depending on the genetic background. Thus, HMDC provides a genotype level overview of mouse models that can help researchers find the most appropriate model for their disease of interest.

## Gene Expression Database (GXD)

The mouse Gene Expression Database (GXD) collects and integrates different types of gene expression information, with a focus on endogenous gene expression in wild-type and mutant mice. Because GXD is an integral component of the larger MGI system, its expression data are combined with the genetic, functional, and phenotypic data collected by MGD. This enables many biologically and biomedically relevant search capabilities and facilitates insights into the molecular mechanism of health and disease. Here, we provide a brief update on GXD data content and then highlight some of the new interface features made possible by the high level of data integration in MGI.

### Classical types of expression data

For many years, GXD has collected mouse developmental expression data from RNA in situ hybridization, immunohistochemistry, in situ reporter (knock-in) RT-PCR, northern blot, and western blot experiments (Finger et al. 2017; Ringwald et al. 1999; Smith et al. 2015). These data are acquired through systematic curation of the scientific literature and by collaborations with large-sale expression projects (Diez-Roux et al. 2011; Harding et al. 2011; Koscielny et al. 2014; Magdaleno et al. 2006; Visel et al. 2004). Curators annotate these data in detail using official gene, mouse strain and allele nomenclature, controlled vocabularies, and an extensive anatomy ontology (Hayamizu et al. 2015), thus enabling data integration and search capabilities. Expression images are included in expression records when available. See http://www.informatics.jax.org/assay/MGI:6147780 for an immunohistochemistry record. As of June 15, 2021, GXD contains detailed expression data for over 15,400 genes, including data from numerous strains of wild-type mice and from > 6600 mutants. GXD now holds > 418,000 images and > 1.83 million expression results from classical types of expression data.

### RNA-Seq and microarray expression experiments

#### Searchable index of RNA-Seq and microarray expression experiments

We recently extended GXD to capture information about RNA-Seq and microarray expression experiments. It remains difficult to find specific expression experiments in the Gene Expression Omnibus (GEO) (Clough and Barrett 2016) and ArrayExpress (Athar et al. 2019) data archives based on biological parameters because sample annotations use free-form metadata provided by data submitters. Therefore, we generated a standardized, searchable index of RNA-seq expression and microarray experiments in ArrayExpress and GEO to help researchers quickly and reliably find data sets of interest (Smith et al. 2020a). We evaluated all mouse microarray and RNA-seq expression data sets in ArrayExpress (including thousands of experiments imported from GEO); identified those that reported endogenous gene expression in wild-type and mutant mice (in keeping with GXD’s scope but including data from postnatal mice as well); and used ontologies, controlled vocabularies, and standard genetic nomenclature to annotate sample metadata (i.e., tissue, age, sex, strain, mutation carried) for each experiment. We also indicated experimental categories (baseline tissue study, wild-type versus mutant comparison) and experimental variables for these experiments. As of June 18, 2021, we have evaluated 16,983 mouse microarray or RNA-seq data sets (including bulk and single-cell RNA-seq data), identified 3199 data sets as relevant for GXD, and completed the metadata annotation for 3188 data sets.

The searchable index is available via the RNA-Seq and Microarray Experiment Search (http://www.informatics.jax.org/gxd/htexp_index). It is current with regard to experiments in ArrayExpress. As ArrayExpress no longer imports experiments from GEO, we are implementing additional procedures to obtain experiments directly from GEO. Our goal is to have a complete and non-redundant representation of RNA-Seq and microarray expression experiments from both repositories.

#### Curation and integration of RNA-seq expression data

The EBI Expression Atlas project selects high-quality RNA-seq data sets from ArrayExpress and GEO and uses a standardized processing pipeline, starting from the primary data, to generate consistently processed transcript per millions (TPM) values (Papatheodorou et al. 2020). We have imported these TPM level data from the Expression Atlas for those RNA-seq experiments that are within GXD’s scope (as determined by our indexing work described above). We have processed these data further to enable their full integration into GXD. Taking advantage of our sample metadata annotations (described above), we identify the unique biological replicate sets for each experiment; determine the averaged quantile-normalized TPM value for each gene per biological replicate set; and assign each of these TPM values a Present/Absent call, using the TPM range bins employed by the Expression Atlas (Baldarelli et al. 2021). Because GXD uses Present/Absent annotations for classical expression assays, this last step permits the full integration of RNA-Seq data with classical types of expression data in database searches, filters, and displays. To date, we have loaded expression data for 70 RNA-Seq experiments in the GXD metadata index. These include 1846 distinct samples that were condensed to 631 biological replicate sets, representing 88 distinct anatomical structures and 85 distinct mouse strains. Mouse genes represented in Expression Atlas RNA-Seq TPM files cover the entire Ensembl transcriptome (nearly 55,000 genome features), including protein-coding and non-coding RNA genes. Total RNA-Seq assay results amount to nearly 35 million, with comprehensive genome coverage for each experiment. While our literature curation effort focuses on mouse development, we have always accepted postnatal data from electronic submission. However, it is worth noting that the incorporation of RNA-Seq data has led to a large increase in postnatal expression data in GXD. As the RNA-Seq data are fully integrated with classical types of expression data, they are accessible through all the search, filtering, and display tools that GXD provides.

### New interface features: searching and analyzing expression data in the larger context

The GXD Home Page (http://www.informatics.jax.org/expression.shtml) provides the best entry point to all the features and resources provided by GXD. Graphical tiles provide a quick overview of, and access to, GXD's search functions. For first time users, a one-page flow chart describes the GXD interface. A Highlights section alerts users of newly added features and data. Additional graphical links are provided to more information (About GXD), to guidelines for electronic data submissions (Fast Track Your Data), to current data content (GXD Statistics), and to Contact information. Tabbed fields at the bottom provide information about new features, curation policies, access to Help documentation, and Links to other resources.

GXD’s interface utilities have been described previously (Finger et al. 2015, 2017). Here, we focus on search and display features that take advantage of the close integration of expression data with genetic, functional, and phenotypic information in MGD/MGI.

#### Expression data and image search

GXD’s most versatile search form not only supports basic expression queries such as ‘Where and when is a given gene expressed?’ or ‘What genes are expressed in a specific tissue?’ but it also enables much more complex searches such as ‘What genes located in a specified genomic interval are expressed in a specific tissue?,’ thus supporting candidate gene queries, or facilitating insights into cis-regulation of gene expression. Further, one can search for expression data of sets of genes defined by Function, Phenotype, or Disease association. For example, one can search for genes that are associated with ‘spina bifida’ and expressed in the ‘neural tube’; or for genes that are involved in ‘signal transduction’ and expressed in the ‘eye’ of ‘Pax6-mutant’ mice. These diverse search parameters can be used to quickly find genes of specific research interest.

#### Developmental anatomy browser

**Fig. 3:**
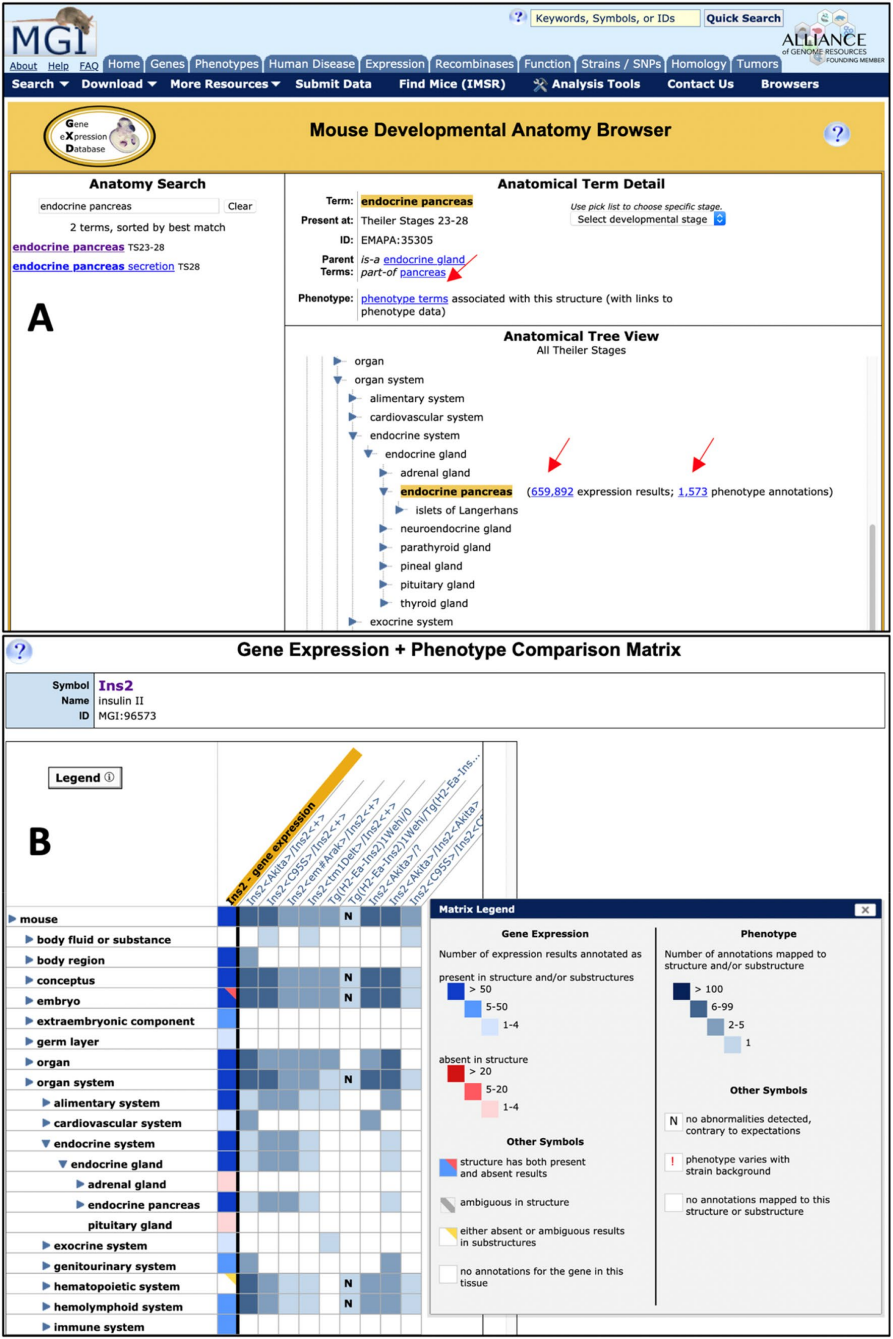
Anatomical comparison of expression and phenotype data. **A** Comparing expression and phenotype data for a specific anatomical structure. The Mouse Developmental Anatomy Browser allows users to search for anatomical structures and to look up the expression and phenotype data associated with these structures. ‘Endocrine pancreas’ is shown as example. Using the Tree View (lower right), one can explore the anatomy by expanding and collapsing the hierarchy. The links indicated by arrows in the Tree View section lead to the expression and phenotype data associated with the selected anatomical structure and its substructures. The link (arrow) in the Term Detail section (upper right) leads to the Mammalian Phenotype (MP) browser, listing the MP terms mapped to the selected anatomical structure, and phenotype data associated with these MP terms. **B** Comparing expression and phenotype pattern for a specific gene. The Gene Expression + Phenotype Matrix displays the expression and phenotype data for a selected gene in the same anatomical matrix view. The gene *Ins2* is shown as an example, with the ‘endocrine system’ expanded along the anatomy axis. The wild-type expression pattern of *Ins2* is displayed in the first column (gold header), the following columns show the anatomical structures phenotypically affected in different *Ins2* mutant mice (different *Ins2* alleles). The coloring of the matrix cells gets progressively darker as the number of expression and phenotype annotations increases; the conventions are defined in the matrix legend (inset)

This browser, used to search for anatomical structures and to look up the associated expression data, has been enhanced to display phenotype data associated with corresponding structures as well (Smith et al. 2019). Thus, it is now easy to compare the expression and phenotype data for specific anatomical structures (Fig. 3A).

#### Gene expression + phenotype comparison matrix

This interactive matrix views, accessible from the phenotype and expression ribbons on MGI gene detail pages, allows users to compare gene expression and phenotype data for a given gene (Fig. 3B). Based on our previously developed Tissue-by-Gene expression matrix views, the Gene Expression + Phenotype Comparison Matrix displays both expression and phenotype data in the mouse developmental anatomy framework, visually juxtaposing the tissues where a gene is normally expressed against tissues where mutations in that gene cause abnormalities. The anatomy axis of the view can be expanded and collapsed, allowing users to explore correlations between gene expression and phenotype at different levels of detail.

#### Expanded search summaries with enhanced filtering capabilities

**Fig. 4:**
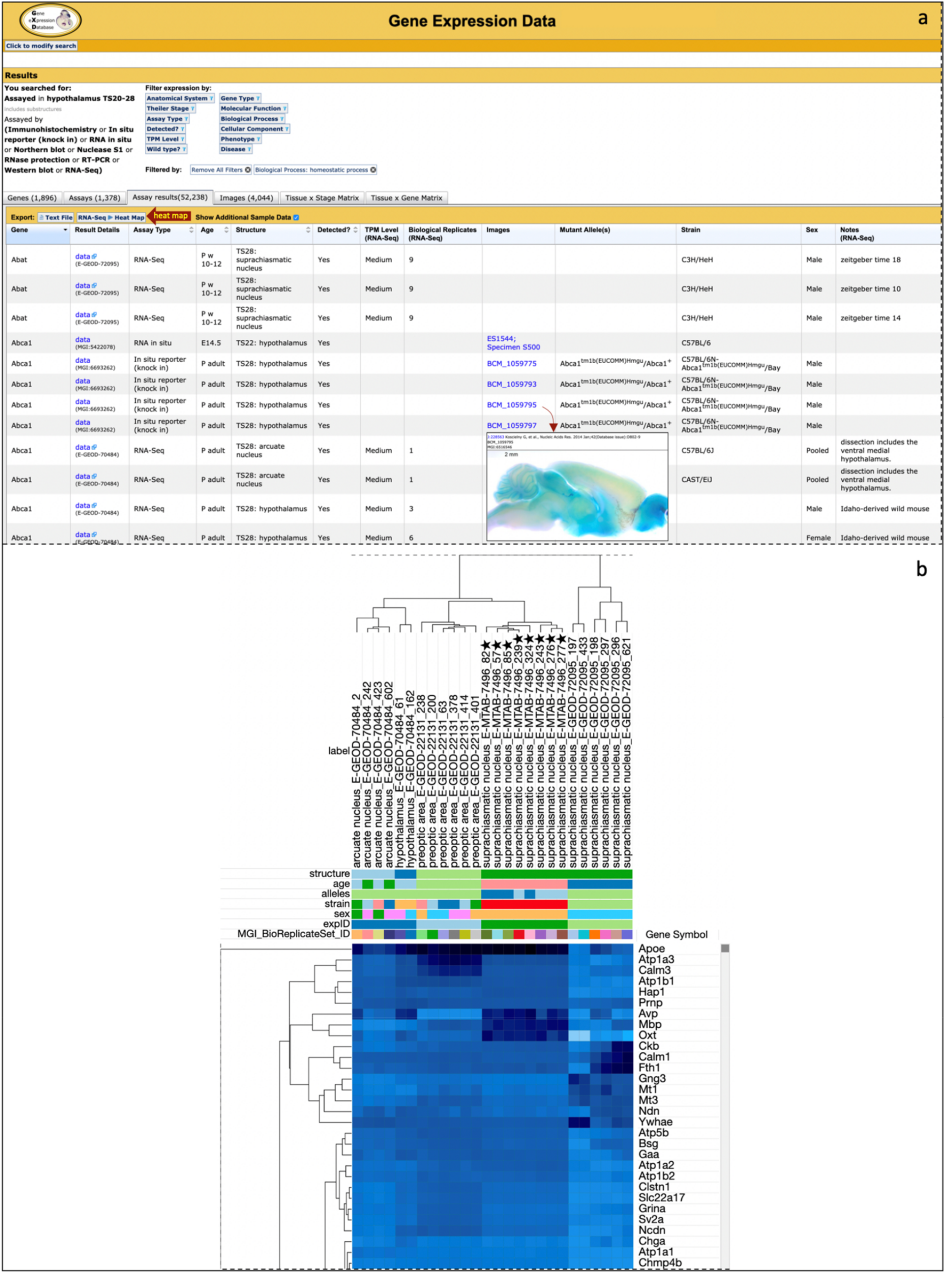
Hypothalamus expression results in GXD for genes involved in homeostasis. **A** Partial search result set shown from a search for expression data assayed in mouse hypothalamus and then filtered for genes annotated to GO Biological Process term: homeostatic process. Classical GXD expression data are integrated with RNA-Seq data via curated source metadata. Assay-level filters allow results to be narrowed by selected sample metadata fields (Anatomical System, Theiler Stage, Assay Type and whether mutant or wild-type mice were assayed). Gene-level filters allow results to be refined by qualitative (Detected?) and quantitative (TPM Level) expression. Gene-level filters also refine results to sets of genes annotated to biological systems of interest (Gene Ontology, Mammalian Phenotype Ontology, Disease Ontology). Expression results are organized into separate views/tabs. The default Assay Results view (shown truncated at the dotted line) displays sample-level metadata for each assayed gene (assay reference column not shown). For classical expression assay types, links in the images column lead to complete assay details including thumbnails of corresponding images, from which details for each image can be accessed (insert shows image detail from (Koscielny et al. 2014)). For RNA-Seq data, a row represents the consolidation of biological replicates, and TPM Level bins are shown for each gene, based on the average, quantile-normalized TPM values from corresponding biological replicate samples with the number of biological replicate samples provided. Clicking the RNA-Seq Heat Map button (red heat map arrow) renders a Morpheus RNA-Seq heat map of the query results. Additional views/tabs for the expression result set (not shown) feature search results organized by Genes, Assays, Images, or two matrix views of the data (Tissue x Stage matrix and Tissue x Gene matrix). Selected filters affect results on all display tabs. **B** RNA-Seq heat map of GXD results from the filtered query rendered with Morpheus (partial heat map shown). Columns represent distinct sample bioreplicate sets and are labeled by a combination of anatomical structure, experiment ID, and bioreplicate set ID (stars represent mutant samples). Metadata shared between samples are indicated by distinct colors in the metadata rows above the gene rows. Colored cells in gene rows reflect average, quantile-normalized TPM values for corresponding columns using a TPM value color scheme that accounts for an expansive dynamic TPM range (dark blue (high) to light blue (low), gray = below threshold, not present in the section of the heat map shown). Genes and samples were clustered using the Hierarchical Clustering feature of Morpheus (Euclidean distance, complete linking). Although the heat map is cropped to show only a few of the 1,893 genes in the result set, both gene-level and sample-level clustering are evident. Notably, the mutant samples (stars) cluster from the wild-type samples and are derived from mice with a mutation in the *Sox2* gene (*Sox2*^*tm1.1Lan*^, ArrayExpress: E-MTAB-7496, (Cheng et al. 2019)

As illustrated at www.informatics.jax.org/mgihome/GXD/FirstTimeUsers.shtml, all of GXD’s expression data search forms lead to the same multi-tabbed displays that summarize data at different levels of detail: Genes, Assays, Assay Results, Images, and in the form of two different Matrix Views (Tissue × Stage and Tissue × Gene). With the incorporation of RNA-Seq data, search summaries have become much more voluminous. We have, therefore, added new data columns to these summaries, supporting new sorting options, and we have added many new filtering capabilities. New sortable data columns include information about TPM-Level (for RNA-Seq data), Strain, and Sex. New filters have been added to filter RNA-Seq expression results by TPM-Level and to filter gene sets based on their gene type (protein-coding, non-coding RNA, etc.); based on high-level Mammalian Phenotype (MP) and Disease Ontology (DO) categories (Bello et al. 2018; Smith and Eppig 2012); and according to their molecular functions, the biological processes they are involved in, and the cellular components in which they are found (high-level Gene Ontology (GO) categories (The Gene Ontology 2019). As illustrated in Fig. 4A, these new filters can be combined with each other and with the expression-related filters that we developed previously (anatomical system, developmental stage, assay type, detected (yes/no), etc.). This provides researchers with new and powerful means to quickly and efficiently narrow down and analyze expression data according to their specific interests, or as a more general exploratory tool.

#### Further analysis of RNA-Seq expression results using Morpheus

One can now employ GXD’s search tools and filters to create customized RNA-Seq data sets (as described above) and, by merely clicking a button, export these data into Morpheus (Fig. 4B). Morpheus, a versatile heat map visualization and analysis tool developed at the Broad Institute (https://software.broadinstitute.org/morpheus/), offers myriad utilities for further display and analysis, including sorting, filtering, hierarchical clustering, nearest neighbor analysis, and visual enrichment. The export function transmits GXD’s sample annotations to Morpheus, where they can be readily used for sorting and filtering.

## Summary

Virtually all advances in medicine rely on the use of animal models to generate, test, and evaluate hypotheses relevant to human biology. As the cost of genome-scale sequencing continues to decrease and new technologies for genome editing become widely adopted, the laboratory mouse is more important than ever as a model system for understanding the biological significance of human genetic variation and for advancing the emergence of genomic medicine. In this era of genome-scale, data-driven biomedical research, the knowledgebases that are part of the MGI environment play a pivotal role in standardizing, integrating, and disseminating information about the laboratory mouse as a model system for understanding human biology and disease processes. MGI’s focus on annotation and nomenclature standards ensures the broadest possible impact of the resource in contributing to the development of new tools and hypotheses for advancing our understanding of the human genome and how it functions and for advancing genomic approaches to improving human health.

## Data Availability

MGI is freely available and accessible from http://www.informatics.jax.org/. Proposed new terms, definitions, synonyms, and changes to the Mammalian Phenotype (MP) Ontology are tracked in GitHub (https://github.com/obophenotype/mammalian-phenotype-ontology/issues). MP ontology files, Adult Mouse Anatomy (MA), and Mouse Developmental Anatomy (EMAPA) ontology files are available in multiple formats from the OBO Foundry (www.obofoundry.org), Ontobee (www.ontobee.org), Ontology Lookup Service (OLS, www.obofoundry.org), and Bioportal (bioportal.bioontology.org). MP and MA ontology files are also available on the MGI reports download site (http://www.informatics.jax.org/downloads/reports/MPheno_OBO.ontology and http://www.informatics.jax.org/downloads/reports/adult_mouse_anatomy.obo).

## Code availability

All source code for the MGI system is available at https://github.com/mgijax.
